# Seasonal succession of ciliate *Mesodinium* spp. with red, green, or mixed plastids and their association with cryptophyte prey

**DOI:** 10.1038/s41598-018-35629-4

**Published:** 2018-11-21

**Authors:** Goh Nishitani, Mineo Yamaguchi

**Affiliations:** 1 Graduate School of Agricultural Science, Tohoku University, Aoba 468-1, Aramaki, Aoba-ku, Sendai 980-0845 Japan; 2 School of Marine Biosciences, Kitasato University, 1-15-1 Kitasato, Minami-ku, Sagamihara, Kanagawa 252-0373 Japan

## Abstract

*Mesodinium* spp. are commonly found in marine and brackish waters, and several species are known to contain red, green, or both plastids that originate from cryptophyte prey. We observed the seasonal succession of *Mesodinium* spp. in a Japanese brackish lake, and we analysed the origin and diversity of the various coloured plastids within the cells of *Mesodinium* spp. using a newly developed primer set that specifically targets the cryptophyte nuclear 18S rRNA gene. *Mesodinium rubrum* isolated from the lake contained only red plastids originating from cryptophyte *Teleaulax amphioxeia*. We identified novel *Mesodinium* sp. that contained only green plastids or both red and green plastids originating from cryptophytes *Hemiselmis* sp. and *Teleaulax acuta*. Although the morphology of the newly identified *Mesodinium* sp. was indistinguishable from that of *M. rubrum* under normal light microscopy, phylogenetic analysis placed this species between the *M. rubrum/major* species complex and a well-supported lineage of *M. chamaeleon* and *M. coatsi*. Close associations were observed in cryptophyte species composition within cells of *Mesodinium* spp. and in ambient water samples. The appearance of suitable cryptophyte prey is probably a trigger for succession of *Mesodinium* spp., and the subsequent abundance of *Mesodinium* spp. appears to be influenced by water temperature and dissolved inorganic nutrients.

## Introduction

Ciliates belonging to genus *Mesodinium* are widely distributed and are abundant in marine and brackish waters^1,2^. The most common species, *Mesodinium rubrum* Lohmann 1908 (previously named as *Myrionecta rubra* Jankowski 1976), causes red water blooms in many coastal ecosystems. Although *M. rubrum* is known as a nontoxic species^3^, blooms of the ciliate can be potentially harmful to aquaculture industries^4,5^. *M. rubrum* is reported to photosynthesize by sequestering the nucleus of its cryptophyte prey, in order to maintain stolen plastids and other organelles^6^. Therefore, the genus *Mesodinium* plays an important role in linking cryptophycean prey and diverse predators in the aquatic microbial food web. For example, the dinoflagellates *Dinophysis* spp., which are a predator of *M. rubrum* and the source of their cryptophyte-derived plastids, have been frequently observed to be precede or to coincide with high densities of *M. rubrum* in time and space^7–10^.

Currently, six marine species of *Mesodinium* have been described and are grouped based on nutritional mode: plastidic (*M. chamaeleon*, *M. coatsi*, *M. major*, and *M. rubrum*) or heterotrophic (*M. pulex* and *M. pupula*). There is some debate as to whether the nutritional mode of plastidic *Mesodinium* species is phototrophic (permanent plastid) or mixotrophic^11,12^. Among the plastidic species, wild *M. major* and *M. rubrum* populations possess red plastids belonging to genera *Teleaulax*, *Plagioselmis*, and *Geminigera*^13–15^, while wild *M. chamaeleon* and *M. coatsi* populations normally contain green plastids^16–18^. Under laboratory conditions, *M. chamaeleon* and *M. coatsi* were maintained by providing the green cryptophyte prey, *Chroomonas vectensis* and *Chroomonas* sp., respectively^17,18^. In another case, *M. chamaeleon* grew best with a red cryptophyte, *Storeatula major*, in the laboratory experiment^19^. Therefore, the availability of suitable cryptophyte prey is important for bloom formation of plastidic *Mesodinium* species.

**Figure 1:**
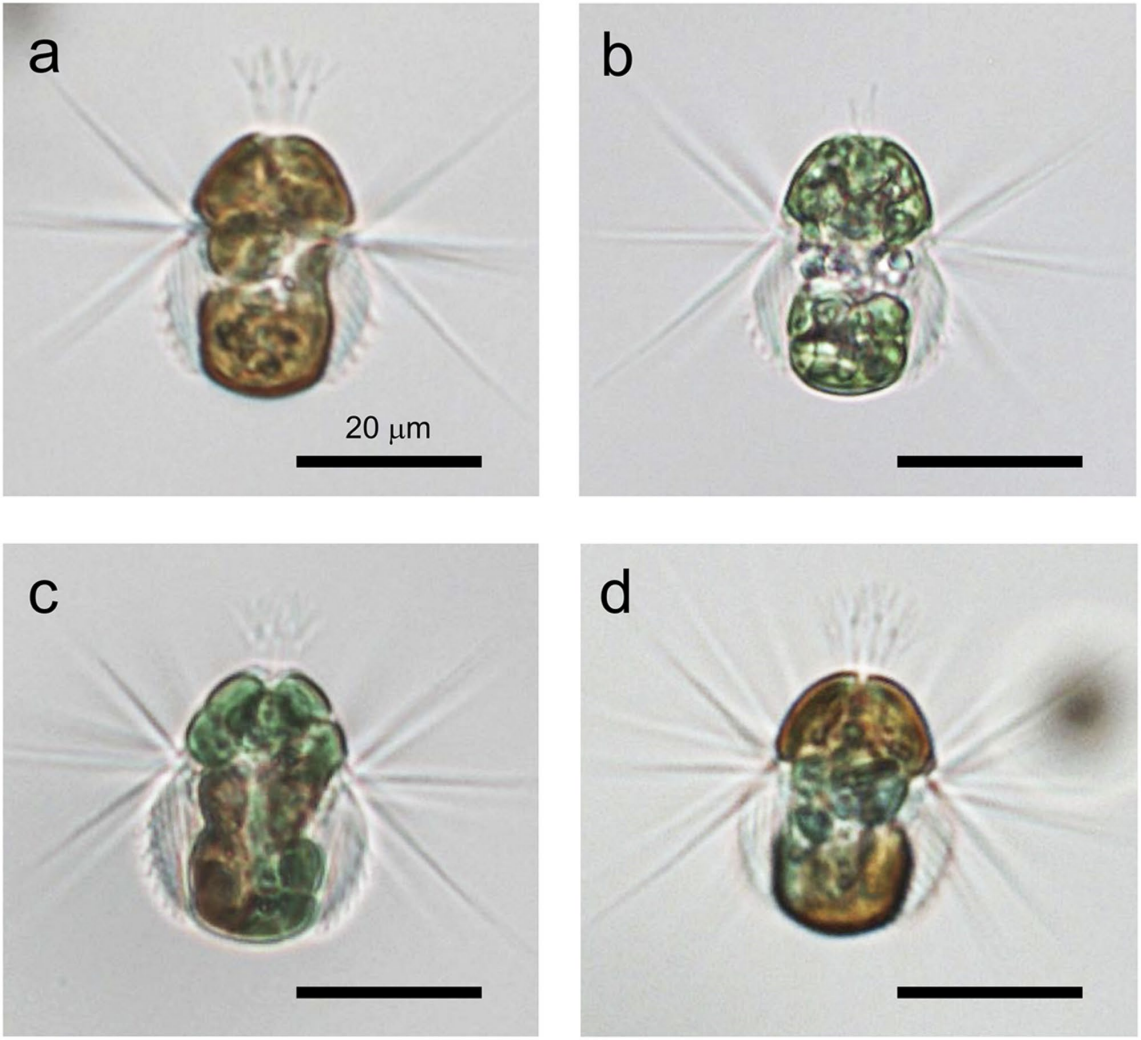
Cells of *Mesodinium* spp. collected from the brackish water of Lake Koyama, Japan. *Mesodinium* contained red (**a**), green (**b**), or red and green (**c**,**d**) plastids derived from cryptophyte prey. All scale bars = 20 μm.

We recently noticed that *Mesodinium* spp. appearing in a Japanese brackish lake possess red or green plastids, or a combination of both (Fig. 1), and the ratio of coloured plastids varied depending on season. This phenomenon poses two intriguing questions: (i) the identify of the originating cryptophyte species of the plastids, and (ii) whether the different coloured *Mesodinium* are the same species. To date, although there are many studies on cultivating *Mesodinium* using cryptophyte prey under laboratory conditions, few studies have documented cryptophyte diversity and the associations between cryptophytes and *Mesodinium* in the field. Herfort *et al*.^14^ investigated cryptophyte diversity in water samples and within cells of *Mesodinium* using a universal primer set targeting the nuclear 18S rRNA gene. Although sequences of the 18S rRNA gene are abundantly available in the GenBank database, this assay is not specific to cryptophyte species and also amplifies diatoms and dinoflagellates. Molecular detection using cryptophyte-specific primer sets targeting the nucleomorph 18S rRNA gene, the plastid *rbcL* gene, and the nuclear 28S rRNA gene has also been reported^13,15,20^. However, these regions are more difficult to identify in cryptophyte species because compared to the nuclear 18S rRNA gene, there are few sequences in the GenBank database.

Therefore, in this study, we first developed a new primer set that specifically amplifies part of the cryptophyte nuclear 18S rRNA gene. This primer set has two advantages: (i) it possesses specificity to cryptophytes and (ii) it produces amplification products that can be aligned with database sequences for species identification of cryptophytes. Using the cryptophyte-specific primer set, we analysed the origin of cryptophyte plastids and their diversity within the cells of *Mesodinium* and also the diversity of cryptophytes in water samples. We also conducted species identification of *Mesodinium* spp. using *Mesodinium*-specific primer sets. Finally, these results were used to deduce the prey preference of *Mesodinium* and the role of cryptophytes on the succession of *Mesodinium* species in the field.

## Results

### Environmental data and seasonal succession of *Mesodinium* populations

**Figure 2:**
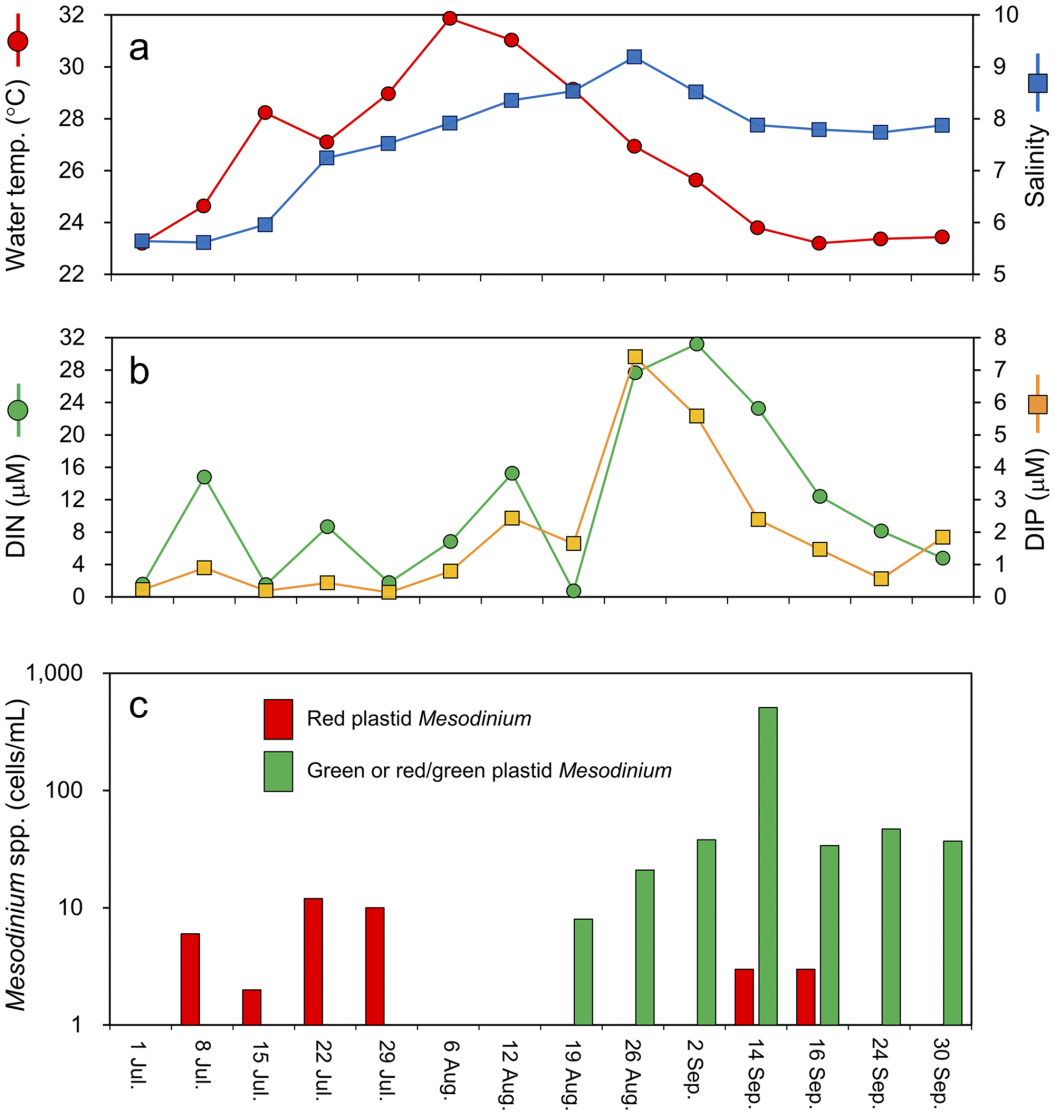
Seasonal changes in environmental factors and cell density of *Mesodinium* spp. in the brackish lake from July to September 2015. (**a**) Water temperature and salinity, (**b**) DIN and DIP, and (**c**) cell density of *Mesodinium* spp. for red plastid-containing *Mesodinium* or for *Mesodinium* with other plastid types were determined throughout the sampling season.

Environmental data, including water temperature, salinity, and dissolved inorganic nutrients (DIN and DIP) are shown in Fig. 2. During the sampling period, water temperature ranged from 23.2 to 31.9 °C, and salinity ranged from 5.6 to 9.2. Water temperature gradually increased from the start of the sampling (1 July) to the maximum value on 6 August and then decreased. No marked variation was observed in salinity with a slight increase toward to the end of the sampling period. DIN ranged from 0.42 to 31.22 μM and DIP ranged from 0.07 to 7.40 μM. Dissolved nutrients were relatively low in the first half of the sampling period and increased in the latter half.

In this brackish lake, the presence of *M. rubrum*-like ciliates was confirmed, and they had three different plastid patterns based on colour: red plastids (Fig. 1a), green plastids (Fig. 1b), or both red and green plastids (Fig. 1c,d). An increase in red-coloured *Mesodinium* was first observed at the start of the sampling period, and then the population was replaced by green and mixed-coloured *Mesodinium* in the latter half (Fig. 2). The maximum cell density of red-coloured *Mesodinium* was 12 cells/mL on 22 July, while the green and mixed-coloured *Mesodinium* reached 510 cells/mL on 14 September. Re-occurrences of red-coloured *Mesodinium* were observed on 14 and 16 September although the densities were relatively low (3 cells/mL). A statistically significant correlation was observed between the cell density of the green and mixed-coloured *Mesodinium* and DIN concentration: r = 0.42 (P = 0.028).

### Genetic analyses of cryptophyte diversity within the cells of *Mesodinium* spp. and in water samples

**Figure 3:**
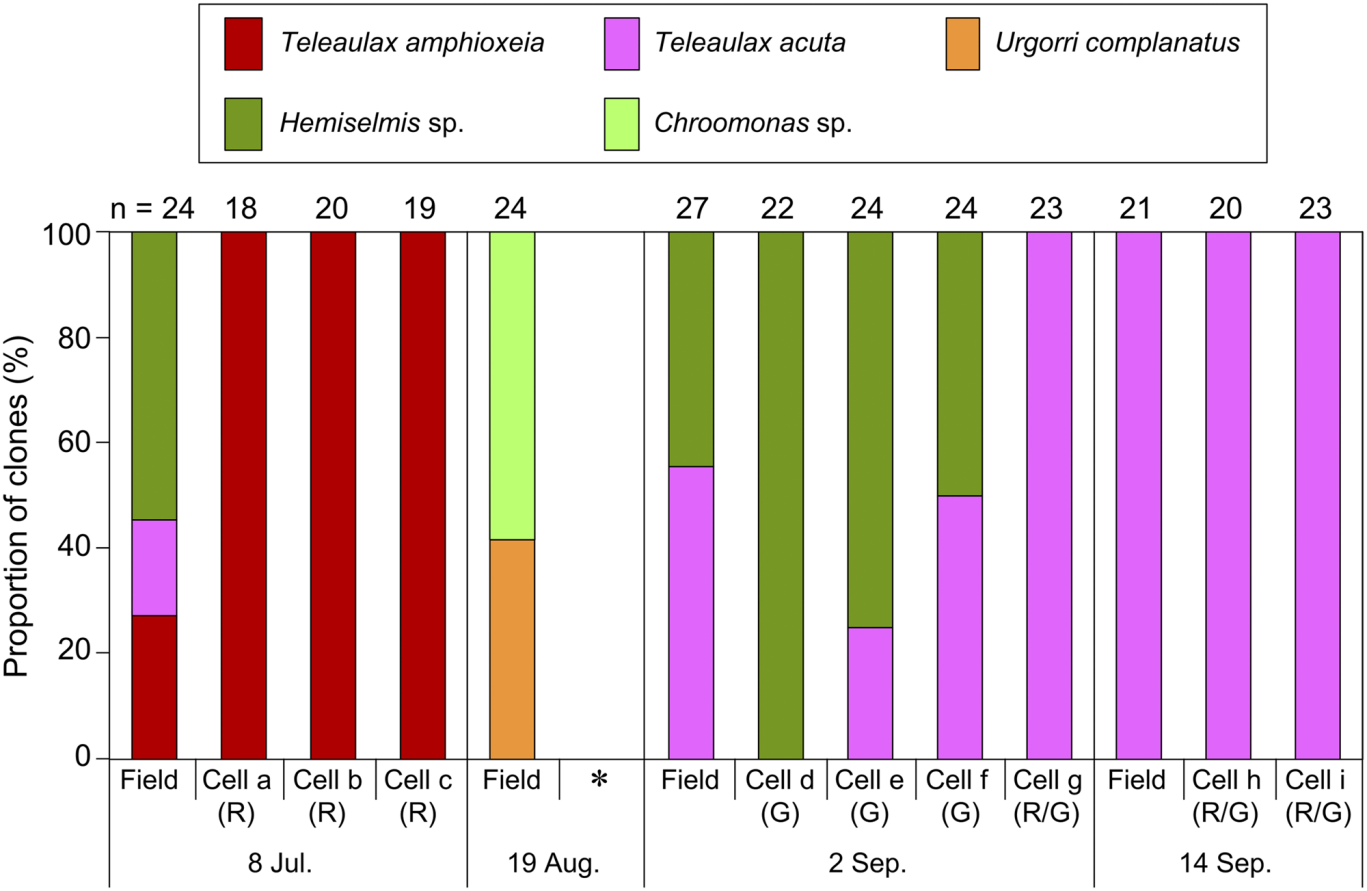
Species distribution of cryptophyte clones recovered from the nine cells of *Mesodinium* (cells **a**–**i**) and the water samples (Field), based on the partial nuclear 18S rRNA gene sequence. On 19 August, no *Mesodinium* cells could be isolated (indicated by the asterisk). Plastid colour is represented by red (R), green (G), or red and green (R/G). The “n” indicates the number of sequenced clones.

A total of 193 cryptophyte sequences were determined from nine cells of *Mesodinium*, and a total of 96 cryptophyte sequences were identified from water samples by cloning (Fig. 3).

The three cells of *Mesodinium* (a, b, and c) collected on 8 July, the beginning of the sampling period, had red-colour plastids and all 57 obtained sequences were of *T. amphioxeia*. However, the filter sample from that date included three cryptophytes species, *T. amphioxeia*, *T. acuta* and *Hemiselmis* sp. On 18 August, no *Mesodinium* cells could be isolated due to the scarcity of cells, and the cryptophyte sequences recovered from the water sample included *Urgorri complanatus* and *Chroomonas* sp. The cryptophyte *U. complanatus* is a red-tide-forming species in brackish waters, with the red plastid colour due to the presence of phycoerythrin^21^, while *Chroomonas* species have green plastids due to the presence of phycocyanin^22^. On 2 September, of the four cells of *Mesodinium* (d, e, f, and g) that were isolated, three cells (d, e, and f) had only green-colour plastids, and one cell (g) had both red and green plastids. In the 93 cryptophyte sequences recovered from four cells of *Mesodinium*, the sequences of *T. acuta* and *Hemiselmis* sp. but not *T. amphioxeia* were recovered. The sequence of *Hemiselmis* sp. showed 98.7% similarity to that of *H. cryptochromatica*, which possesses green plastids^22^. Cryptophyte diversity within the cells of *Mesodinium* was relatively well consistent with that in the water sample collected on the same day. On 14 September, two cells of *Mesodinium* had mix-coloured (red and green) plastids, although both cells and the water sample only had cryptophyte sequences of *T. acuta*.

### Genetic analysis of nine cells of *Mesodinium* for species identification

Although the morphology of nine cells of *Mesodinium* that we isolated was consistent with that of *M. rubrum*, the period of occurrence and plastid characteristics were clearly different. Therefore, we confirmed the species of all nine cells of *Mesodinium* by sequencing the nuclear 18S rRNA gene sequences. Three cells (a, b, and c) that were isolated in the beginning of the sampling period were identical to the sequences of *M. rubrum* (variant B) catalogued as AB364286 in GenBank. The sequences of the remaining six cells (d, e, f, g, h, and i) were identical to each other but were not identical with any variants in *M. rubrum* or environmental sequences catalogued in GenBank.

**Figure 4:**
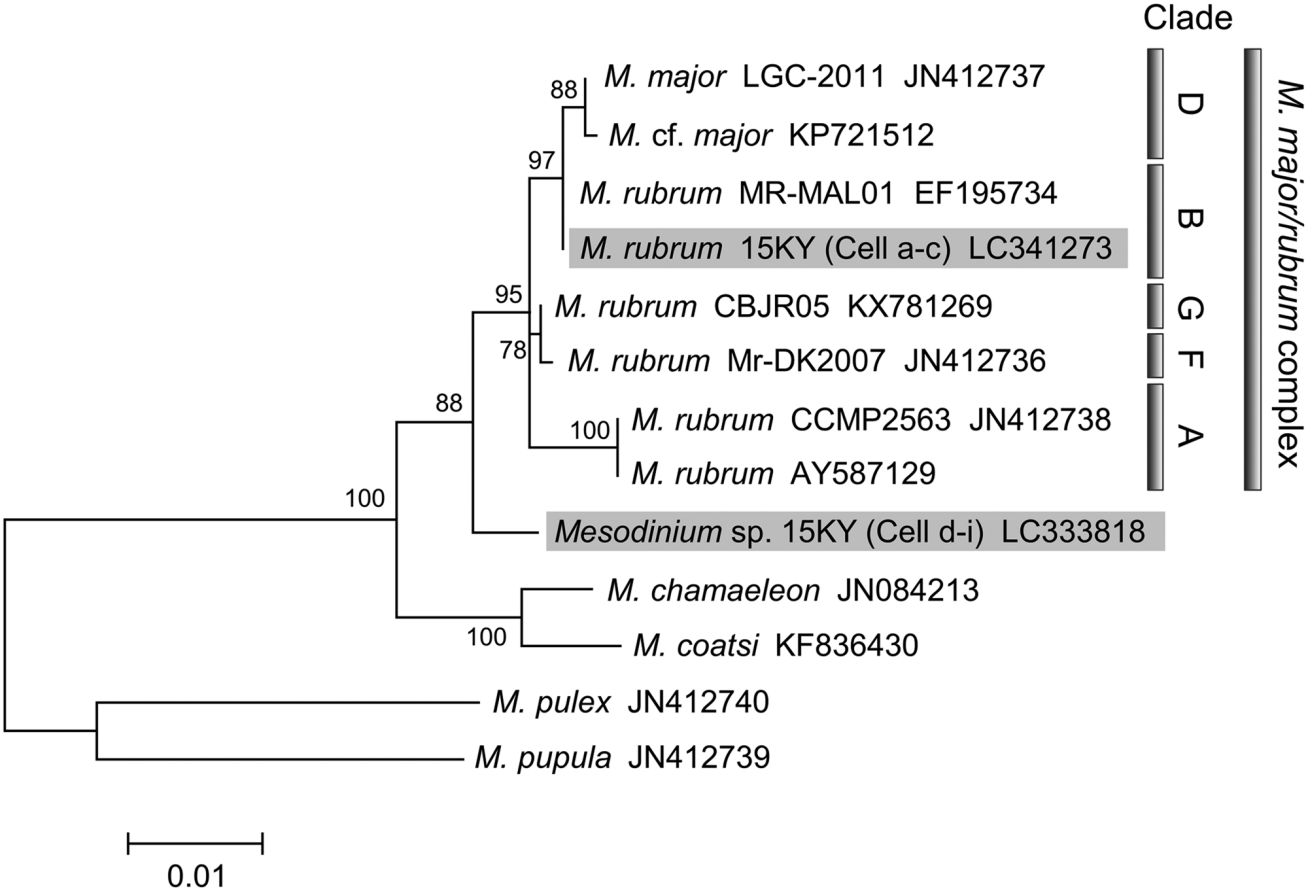
Unrooted maximum likelihood phylogenetic tree of the nuclear 18S rRNA gene of *Mesodinium* spp. analysed in this study together with sequences from the GenBank database. Sequences determined in this study are highlighted in gray. Clades (A, B, D, F, and G) are as referenced by Herfort *et al*.^14^ and Johnson *et al*.^20^ within the *M. major/rubrum* complex. Bootstrap values of >50% on the tree produced using MEGA software ver. 7 are given as the percentage of 100 bootstrap replicates at the respective nodes. The final dataset contains 1,218 informative sites. The scale bar represents the number of substitutions per site.

We tentatively identified the novel sequence as *Mesodinium* sp., and the sequence had 15-22/1483 bp differences (similarity: 98.5–99.0%) with those of variants in *M. rubrum*. The phylogenetic position within the genus *Mesodinium* is represented in Fig. 4. As shown previously^16^, genus *Mesodinium* formed four distinct clades represented by *M. pulex*, *M. pupula*, *M. chameleon/M. coatsi*, and the *M. major*/*rubrum* complex, respectively. Furthermore, the *M. major*/*rubrum* species complex was divided into eight subclades based on 18S rRNA gene fragment, the complete internally transcribed spacer region, and a partial region of the 28S rRNA gene^14,16,20^. At present, nearly complete 18S rRNA gene sequences are available in five (variants A, B, D, F, and G) out of the eight subclades. The novel sequence of *Mesodinium* sp. analysed in this study was not grouped with any variants of the *M. major*/*rubrum* species complex on the phylogenetic tree, which had 88% bootstrap support (Fig. 4).

## Discussion

Here, we showed that the newly observed *Mesodinium* is not as a variant of *M. rubrum* but a novel species “*Mesodinium* sp.”. While this species could not be distinguished from *M. rubrum* based on morphology under normal light microscopy, it could be clearly differentiated based on ecology, especially plastid origin. Previous field investigations revealed that natural cells of *M. rubrum* possess plastids originating from cryptophyte *Teleaulax amphioxeia*^13,14^. Furthermore, to date, all stable cultures of *M. rubrum* have been established using either *Teleaulax* or *Geminigera* as prey^23–28^, both of which are closely related and have red plastids that contain phycoerythrin biliprotein. To date, there have been no reports on the culture of *M. rubrum* with green-plastid cryptophytes as a prey. We also tried to cultivate *Mesodinium* sp. isolated from the brackish lake using filtered sterilized lake water and a culture strain of green-plastid cryptophyte *Chroomonas* sp. as a prey that had been isolated from the same area (Supplementary Table S1). The 18S rRNA gene sequence of the *Chroomonas* sp. was identical to that detected by gene cloning from the field analysis on 19 August. *Mesodinium* sp. could only be maintained for 3 weeks with the green-plastid cryptophyte *Chroomonas* sp. Our genetic analysis (Fig. 3) shows that green-coloured *Mesodinium* sp. possessed the plastid originated from the genus *Hemiselmis* not *Chroomonas*, suggesting that cryptophyte prey may not be suitable for supporting the growth of *Mesodinium* sp. We then attempted to cultivate *Mesodinium* sp. with red-plastid cryptophyte *Teleaulax amphioxeia* (Supplementary Table S1). The culture strain of *Mesodinium* sp. with *T. amphioxeia* was maintained for 1 year and 4 months, but this prey did not seem to be optimal. Given that the DNA of *T. acuta* was detected from the natural cells of *Mesodinium* sp. by genetic analysis in this study, *Mesodinium* sp. may have species-level *Hemiselmis* and *Teleaulax* prey preference for supporting optimal growth, while *M. rubrum* shows *Teleaulax* genus-level selection^28^. Finally, we consider that *M. rubrum*-like ciliates containing green plastids, namely *Mesodinium* sp., are distinct species from *M. rubrum* based on sequence difference and their capability for utilizing plastids from *Hemiselmis* cryptophytes, although further experiments such as observations of ultrastructure and growth physiology are required to confirm the true differentiation of species or as a variant thereof. Yih *et al*. have reported an interesting observation that natural cells of *M. rubrum* collected from Gomso Bay, Korea had different plastid colours (red, green, or a combination)^29^, although they did not analysed the DNA sequences of the cells of *M. rubrum*. Such *M*. *rubrum*-like ciliates possessing green plastids might also have a distinct 18S rRNA gene sequence, as shown in this study.

Our field monitoring and genetic analysis revealed that the seasonal succession from *M. rubrum* to *Mesodinium* sp. occurred in a brackish lake, despite the indistinguishable morphology. The abundance of both species seems to be related to water temperature and dissolved inorganic nutrients. Water temperature during the sampling period (23.2–31.9 °C) in Lake Koyama was higher than the optimum growth temperature of *Mesodinium*, especially when the water temperature exceeded 28 °C. Johnson *et al*. have reported that the highest concentrations (>1000 cells/mL) of *M. rubrum* were observed when the average water temperature in Chesapeake Bay was 18.1 °C^30^. In laboratory experiments, culture experiments of *M. rubrum* are typically conducted in temperatures ranging from 15 to 18 °C^26,31−33^. The dissolved inorganic nutrients (DIN and DIP) may also influence the growth of *Mesodinium*. Tong *et al*. reported that DIN and DIP can be utilized by *M. rubrum* for enhanced growth when requirements for optimal cryptophyte prey are met^33^. Previous studies have found that ingestion of cryptophytes represents less than 10% of the required carbon requirements for *M. rubrum* growth and maintenance^31,34^, indicating the importance of dissolved inorganic nutrients for autotrophic growth of *M. rubrum*. In contrast, *M. chamaeleon* obtains about half of its energy from photosynthesis, and its growth is not affected by dissolved inorganic nutrients^19^. In this study, the increase in cell number of *Mesodinium* sp. observed in the latter half of the sampling period might be attributable to the increased DIN and DIP (Fig. 2), either directly or indirectly (i.e. by stimulating growth of optimal prey). However, further laboratory studies are needed to address the role of nutrients in this novel *Mesodinium* sp.

Sequences of three cryptophyte species, *T. amphioxeia*, *T. acuta*, and *Hemiselmis* sp., were detected in the water sample collected on 8 July, however, only *T. amphioxeia* was detected from three cells of *M. rubrum* collected on the same day. This observation suggests that *M. rubrum* preferentially ingested *T. amphioxeia* as previously reported in the field^13,14^ and laboratory^24,26–28^. On the other hand, *Mesodinium* sp. analysed in this study possessed either red or green plastids, or both, depending on the cryptophyte species present; *M. chamaeleon* showed a similar pattern in laboratory experiments^17,19^. Although the morphology of *Mesodinium* sp. was remarkably similar to that of *M. rubrum*, it seems to resemble *M. chamaeleon* in the plastid utilization.

The growth physiology and plastid replacement of *Mesodinium* sp. under laboratory cultivation is of particular interest when suitable red and green cryptophyte, *T. acuta* and *Hemiselmis* sp., are supplied. On 14 September, *T. acuta* was the only cryptophyte detected in both the water sample and the cells of *Mesodinium* sp., while the cells of *Mesodinium* contained both red and green plastids. Since the nucleus of cryptophyte prey has been shown to be lost over time in *M. rubrum*^6^ and degraded rather quickly in *M. chamaeleon*^17^, any nucleus associated with the green plastids of isolated *Mesodinium* sp. were probably older than the red ones, and their DNA may have been too low to be amplified by PCR.

In conclusion, *M. rubrum* preferentially ingests red plastid cryptophytes, especially *T. amphioxeia*, in the field, while *Mesodinium* sp. analysed in this study utilized both red and green plastid cryptophytes as prey. Although the morphology of the present sample and *M. rubrum* could not be distinguishable under normal light microscopy, the 18S rRNA gene sequences had 15-22/1483 bp differences. The appearance of suitable cryptophyte prey is likely the most important factor for succession of *Mesodinium* spp., and the abundance of *Mesodinium* appears to be influenced by water temperature and perhaps dissolved inorganic nutrients.

## Methods

### Sample collection and water chemistry

Lake Koyama (35°30′N, 134°9′E) in Tottori Prefecture, southwest Japan, is a brackish lake with a mean depth of 2.8 m and is one of the largest lakes in Japan. Water samples were collected from the surface layer from July to September 2015. Water temperature and salinity were measured with a multi-parameter water quality sonde (Hydrolab DS5, OTT Hydromet, Germany). Dissolved inorganic nitrogen (DIN) and dissolved inorganic phosphorous (DIP) were determined using an AutoAnalyzer (TRACCS-2000, BL TEC, Japan) after filtering water samples through a membrane filter (0.45 μm, Millipore, USA).

### Cell enumeration

Live cell density of *Mesodinium* spp. was determined separately for each plastid colour under an inverted microscope (Nikon Eclipse Ti-U, Japan). However, green cells and the cells with both coloured plastids (green/red) were counted together due to the difficulty of distinguishing cells with green only or mixed green and red plastids. Enumeration of cell density in cryptophytes was not conducted in this study.

### Sample collection for DNA analysis

For the DNA analysis, two types of samples were prepared: (i) PCR tubes containing a single cell of *Mesodinium* to analyse cryptophyte prey diversity within the ciliate cell and for species identification of *Mesodinium* and (ii) membrane filters through which water samples were filtered to analyse cryptophyte diversity in surface water. In the single cell analysis, a total of nine cells of *Mesodinium* were isolated by micropipetting on the following dates in 2015: 8 July (cells a, b, and c), 2 September (cells d, e, f, and g), and 14 September (cells h and i). The live cells were washed several times with filtered (0.1 μm pore size) seawater and placed individually in 0.2 mL PCR tubes containing 10 μL of TE buffer (Tris-hydrochloride buffer, pH 8.0, containing 1.0 mM EDTA). In the membrane filter analysis, samples were obtained on the following dates in 2015: 8 July, 19 August, 2 September, and 14 September. Each 50 mL aliquot of water was passed through a plankton net (20 μm mesh size) to remove large organisms and filtered through a membrane filter (25 mm diameter with 1 μm pore size; Nuclepore Track-Etch Membrane, Whatman plc, UK). All PCR tubes and filters were stored at −25 °C until DNA extraction.

### DNA extraction, PCR amplification, and gene cloning

DNA was extracted from single cell samples by heating at 98 °C for 20 min and from filters using the DNeasy Plant Mini Kit (Qiagen, Germany). We have developed a new cryptophyte-specific primer set (n18S-Crypt24F/n18S-Crypt860R; Table 1) that specifically amplifies a portion of the nuclear 18S rRNA gene of cryptophytes and can be used for the analysis of cryptophyte diversity in field samples, as well as within cells of *Mesodinium*. This primer set was designed not to amplify the nucleomorph gene of cryptophytes. The newly designed primer set was shown to be effective in this study because no sequences other than cryptophyte sequences were detected in field and *Mesodinium* samples. First, the cryptophyte diversity within each cell of *Mesodinium* was analysed using DNA from the PCR tubes with the newly developed primer set. Single-cell polymerase chain reaction (PCR) was performed using a Veriti thermal cycler (Thermo Fisher Scientific, USA) with a reaction mixture (20 μL) containing 1.0 μL template DNA, 0.2 mM of each dNTP, 1 × PCR buffer, 1.5 mM Mg^2+^, 1.0 U KOD -Plus- ver. 2 (TOYOBO, Japan, with intensive 3′ → 5′ exonuclease activity), and 0.2 μM of each primer. The PCR amplification conditions were as follows: initial denaturation at 94 °C for 2 min, followed by 32 cycles of 98 °C for 10 s, 56 °C for 30 s, and 68 °C for 60 s. The resulting PCR amplifications were run on 1.5% agarose gels, and gene cloning was then conducted according to Nishitani *et al*.^35^. Second, cryptophyte diversity in each water samples was analysed using DNA from the filters with the same primer set (n18S-Crypt24F/n18S-Crypt860R) and amplification conditions and gene cloning as described above. Finally, the species of the nine cells of *Mesodinium* were identified using DNA from the PCR tubes targeting the nuclear 18S rRNA gene with specific primer sets for *Mesodinium* (three primer sets were designed in this study: n18S-Meso1F/n18S-Meso580R, n18S-Meso470F/n18S-Meso1108R, and n18S-Meso1006F/n18S-Meso1580R; Table 1). These primer sets specifically amplify DNA of genus *Mesodinium* even if cryptophyte DNA is present in the ciliate. PCR amplification conditions were as follows: initial denaturation at 94 °C for 2 min, followed by 34 cycles at 98 °C for 10 s, 60 °C for 30 s, and 68 °C for 45 s. PCR products were then purified using the ExoSAP-IT PCR product cleanup reagent (Thermo Fisher Scientific, USA). The DNA sequences of *Mesodinium* spp. were determined directly, without gene cloning. All sequences obtained in this study were determined using a DYEnamic ET Terminator Cycle Sequencing Kit (GE Healthcare, Little Chalfont, UK) and analysed on a 3730xl DNA Analyzer (Thermo Fisher Scientific, USA).

**Table 1:** Primers used in this study.

Primer name	Sequence (5′-3′)	Annealing site
n18S-Crypt24F	ATTAAGCCATGCATGTCTTAGTG	24-46^a^
n18S-Crypt860R	CTCTGACAACGAAATATRAACGG	838-860^a^
n18S-Meso1F	AACCTGGTTGATCCTGCCAG	1-20^b^
n18S-Meso580R	ACGACGTACAGACTACGGACGT	559-580^b^
n18S-Meso470F	TTCCKATTAGCAACTGATGGGA	470-491^b^
n18S-Meso1108R	GTCATYAGCCTGTCAATCACGC	1087-1108^b^
n18S-Meso1006F	TTGACGGAAGAGCACCATAAGAC	1006-1028^b^
n18S-Meso1580R	TCACCTACGGAAACCTTGTTACG	1558-1580^b^

The annealing sites refer to the sequences of *Teleaulax amphioxeia* AJ007287 (a) and *Mesodinium pulex* DQ845294 (b) in GenBank. All primers were newly developed in this study.

### Phylogenetic analysis

All PCR amplification products were sequenced, and the forward and reverse sequences were aligned using GENETYX software (Genetyx Corporation, Japan). All cryptophyte sequences obtained in this study were checked against GenBank using the nucleotide Basic Local Alignment Search Tool (BLASTN). To deduce the species of *Mesodinium* analysed in this study, sequences of the 18S rRNA gene for *Mesodinium* were aligned and an unrooted phylogenetic tree was generated by MEGA ver. 7 software^36^ using the maximum likelihood (ML) method with default settings. The topology of the phylogenetic tree was evaluated using the bootstrap method, with 100 replicates.

## Electronic supplementary material

Supplementary information

## Data Availability

All sequences obtained in this study were deposited in the DDBJ, EMBL, and GenBank databases (*Mesodinium rubrum*, LC341273; *Mesodinium* sp., LC333818; *Chroomonas* sp., LC334060; *Hemiselmis* sp., LC334059; *Teleaulax acuta*, LC334057; *Teleaulax amphioxeia*, LC334056; and *Urgorri complanatus*, LC334058).
