# Supplementary material for: Seasonal succession of ciliate Mesodinium spp. with red, green, or mixed plastids and their association with cryptophyte prey
Source: Sci Rep. 2018 Nov 21;8:17189. doi: 10.1038/s41598-018-35629-4 (PMC6249236; doi:10.1038/s41598-018-35629-4)
Supplement: Supplementary file 1 — Supplementary information [file 41598_2018_35629_MOESM1_ESM.pdf]

Supplementary information

Seasonal succession of ciliate *Mesodinium* spp. with red, green, or mixed plastids and their association with cryptophyte prey

Goh Nishitani[1,*], **Mineo Yamaguchi**[2,*]

[1] Graduate School of Agricultural Science, Tohoku University, Aoba 468-1, Aramaki, Aoba-ku, Sendai 980-0845, Japan
[2] School of Marine Biosciences, Kitasato University, 1-15-1 Kitasato, Minami-ku, Sagamihara, Kanagawa 252-0373, Japan

[*]ni5@tohoku.ac.jp, mineo@kitasato-u.ac.jp

	Culture period (days)							
**Prey species**	**0**	**2**	**7**	**9**	**13**	**16**	**19**	**22**
Chroomonas sp.	1	2	2	1	1	0		
Chroomonas sp.	1	2	1	3	0			
Chroomonas sp.	1	3	2	5	8	0		
Chroomonas sp.	1	1	8	23	8	0		
Chroomonas sp.	1	1	4	4	13	15	4	0
Chroomonas sp.	1	1	2	2	4	1	0	
Chroomonas sp.	1	1	4	3	16	7	0	
Chroomonas sp.	1	0						
Chroomonas sp.	1	2	4	4	4	0		
Chroomonas sp.	1	2	1		0			
Chroomonas sp.	1	1	2	3	2	0		
Chroomonas sp.	1	1	1	0				
Chroomonas sp.	1	1	1	3	0			
Chroomonas sp.	1	1	9	21	14	1	0	
Chroomonas sp.	1	2	6	8	2	0		
Chroomonas sp.	1	2	2	3	0			
Teleaulax amphioxeia	1	4	20	10				
Teleaulax amphioxeia	1	0						
Teleaulax amphioxeia	1	0						
Teleaulax amphioxeia	1	0						
Teleaulax amphioxeia	1	0						
Teleaulax amphioxeia	1	0						
Teleaulax amphioxeia	1	2	15	0				
Teleaulax amphioxeia	1	1	22	>50	*			
Teleaulax amphioxeia	1	0						
Teleaulax amphioxeia	1	3	6	0				
Teleaulax amphioxeia	1	0						
Teleaulax amphioxeia	1	3	33	>70	*			
Teleaulax amphioxeia	1	4	11	0				
Teleaulax amphioxeia	1	0						
Teleaulax amphioxeia	1	0						
Teleaulax amphioxeia	1	2	24	0				

Table S1 Changes in cell number of *Mesodinium* sp. when cultured with two different cryptophyte prey items; *Chroomonas* sp. (green plastid) and *Teleaulax amphioxeia* (red plastid). All cultures were conducted under the culture condition of 22. 5 °C using 48 well microplates with 0.5 mL of medium in each well. The asterisks indicate that cells of *Mesodinium* were transferred to a 50 mL flask containing 20 mL of new medium for further culturing.
